# Supplementary material for: Reteplase Fc-fusions produced in N. benthamiana are able to dissolve blood clots ex vivo
Source: PLoS One. 2021 Nov 30;16(11):e0260796. doi: 10.1371/journal.pone.0260796 (PMC8631678; doi:10.1371/journal.pone.0260796)
Supplement: S1 Fig — Green: SP; Magenta: mRFP; Blue: CH2-CH3 domain of Fc; Grey: Linker. Potential N-glycosylation sites are in blue, mutations on CH3 domain are in red. Hydrolysis of the PA peptide bond between Arg-Ile (RI, in green) in the kringle-2 domain generates two polypeptide chains. (PDF) [file pone.0260796.s001.pdf]

Fig. S1: Protein sequence of Alteplase (tPA), reteplase (rPA) and rPA fusions. Green: SP; Magenta: mRFP; Blue: CH2-CH3 domain of Fc; Grey: Linker. Potential N-glycosylation sites are in blue, mutations on CH3 domain are in red. Hydrolysis of the PA peptide bond between Arg-Ile (RI, in green) in the kringle-2 domain generates two polypeptide chains

rPA-mRFP

MANKHLSLSLFLVLLGLSASLASGSYQG**N**SDCYFGNGSAYRGTHSLTESGASCLPWNSMILIGKVYTAQNPSAQALGLGKHNYCRNP
DGDAKPWCHVLKNRRLTWEYCDVPSCSTCGLRQYSQPQF**RI**KGGLFADIASHPWQAAIFAKHRRSPGERFLCGGILISSCWILSAAH
CFQERFPPHHLTVILGRTYRVVPGEEEQKFEVEKYIVHKEFDDDTYDNDIALLQLKSDSSRCAQESSVVRTVCLPPADLQLPDWTEC
ELSGYGKHEALSPFYSERLKEAHVRLYPSSRCTSQHLL**N**RTVTDNMLCAGDTRSGGPQANLHDACQGDSGGPLVCLNDGRMTLVGII
SWGLGCGQKDVPGVYTKVTNYLDWIRDNMRPGSASSEDVIKEFMRFKVRMEGSVNGHEFEIEGEGEGRPYEGTQTAKLKVTKGGPLP
FAWDILSPQFQYGSKAYVKHPADIPDYLKLSFPEGFKWERVMNFEDGGVVTVTQDSSLQDGEFIYKVKLRGTNFPSDGPVMQKKTMG
WEASTERMYPEDGALKGEIKMRLKLKDGGHYDAEVKTTYMAKKPVQLPGAYKTDIKLDITSHNEDYTIVEQYERAEGRHSTGA-

rPA-^{H}Fc

MANKHLSLSLFLVLLGLSASLASGSYQG**N**SDCYFGNGSAYRGTHSLTESGASCLPWNSMILIGKVYTAQNPSAQALGLGKHNYCRNP
DGDAKPWCHVLKNRRLTWEYCDVPSCSTCGLRQYSQPQF**RI**KGGLFADIASHPWQAAIFAKHRRSPGERFLCGGILISSCWILSAAH
CFQERFPPHHLTVILGRTYRVVPGEEEQKFEVEKYIVHKEFDDDTYDNDIALLQLKSDSSRCAQESSVVRTVCLPPADLQLPDWTEC
ELSGYGKHEALSPFYSERLKEAHVRLYPSSRCTSQHLL**N**RTVTDNMLCAGDTRSGGPQANLHDACQGD**S**GGPLVCLNDGRMTLVGII
SWGLGCGQKDVPGVYTKVTNYLDWIRDNMRPEPKSCDKTHTCPPCPAPELLGGPSVFLFPPKPKDTLMISRTPEVTCVVVDVSHEDP
EVKFNWYVDGVEVHNAKTKPREEQY**N**STYRVVSVLTVLHQDWLNGKEYKCKVSNKALPAPIEKTISKAKGQPREPQVYTLPPSRDEL
TKNQVSLTCLVKGFYPSDIAVEWESNGQPENNYKTTPPVLDSDGSFFLYSKLTVDKSRWQQGNVFSCSVMHEALHNHYTQKSLSLSP
GK-

rPA-^{L}Fc

MANKHLSLSLFLVLLGLSASLASGSYQG**N**SDCYFGNGSAYRGTHSLTESGASCLPWNSMILIGKVYTAQNPSAQALGLGKHNYCRNP
DGDAKPWCHVLKNRRLTWEYCDVPSCSTCGLRQYSQPQF**RI**KGGLFADIASHPWQAAIFAKHRRSPGERFLCGGILISSCWILSAAH
CFQERFPPHHLTVILGRTYRVVPGEEEQKFEVEKYIVHKEFDDDTYDNDIALLQLKSDSSRCAQESSVVRTVCLPPADLQLPDWTEC
ELSGYGKHEALSPFYSERLKEAHVRLYPSSRCTSQHLL**N**RTVTDNMLCAGDTRSGGPQANLHDACQGD**S**GGPLVCLNDGRMTLVGII
SWGLGCGQKDVPGVYTKVTNYLDWIRDNMRPGGGGSGGGGSLGGPSVFLFPPKPKDTLMISRTPEVTCVVVDVSHEDPEVKFNWYVD
GVEVHNAKTKPREEQY**N**STYRVVSVLTVLHQDWLNGKEYKCKVSNKALPAPIEKTISKAKGQPREPQVYTLPPSRDELTKNQVSLTC
LVKGFYPSDIAVEWESNGQPENNYKTTPPVLDSDGSFFLYSKLTVDKSRWQQGNVFSCSVMHEALHNHYTQKSLSLSPGK-

rPA-mFc

MANKHLSLSLFLVLLGLSASLASGSYQG**N**SDCYFGNGSAYRGTHSLTESGASCLPWNSMILIGKVYTAQNPSAQALGLGKHNYCRNP
DGDAKPWCHVLKNRRLTWEYCDVPSCSTCGLRQYSQPQF**RI**KGGLFADIASHPWQAAIFAKHRRSPGERFLCGGILISSCWILSAAH
CFQERFPPHHLTVILGRTYRVVPGEEEQKFEVEKYIVHKEFDDDTYDNDIALLQLKSDSSRCAQESSVVRTVCLPPADLQLPDWTEC
ELSGYGKHEALSPFYSERLKEAHVRLYPSSRCTSQHLL**N**RTVTDNMLCAGDTRSGGPQANLHDACQGD**S**GGPLVCLNDGRMTLVGII
SWGLGCGQKDVPGVYTKVTNYLDWIRDNMRPGGGGSGGGGSLGGPSVFLFPPKPKDTLMISRTPEVTCVVVDVSHEDPEVKFNWYVD
GVEVHNAKTKPREEQY**N**STYRVVSVLTVLHQDWLNGKEYKCKVSNKALPAPIEKTISKAKGQPREPQVYTLPPSRDELTKNQVSL**R**C
HVKGFYPSDIAVEWESNGQPENNYKTT**K**PVLDSDGSF**R**LYSKLTVDKSRWQQGNVFSCSVMHEALHNHYTQKSLSLSPGK-

tPA- Alteplase (yellow: reteplase)

SYQVICRDEKTQMIYQQHQSWLRPVLRSNRVEYCWCNSGRAQCHSVPVKSCSEPRCFNGGTCQQALYFSDFVCQCPEG
FAGKCCEIDTRATCYEDQGISYRGTWSTAESGAECTNW**N**SSALAQKPYSGRRPDAIRLGLGNHNYCRNPDRDSKPWCY
VFKAGKYSSEFCSTPACSEG**N**SDCYFGNGSAYRGTHSLTESGASCLPWNSMILIGKVYTAQ**N**PSAQALGLGKHNYCRN
PDGDAKPWCHVLKNRRLTWEYCDVPSCSTCGLRQYSQPQF**RI**KGGLFADIASHPWQAAIFAKHRRSPGERFLCGGILI
SSCWILSAAHCFQERFPPHHLTVILGRTYRVVPGEEEQKFEVEKYIVHKEFDDDTYDNDIALLQLKSDSSRCAQESSV
VRTVCLPPADLQLPDWTECELSGYGKHEALSPFYSERLKEAHVRLYPSSRCTSQHLL**N**RTVTDNMLCAGDTRSGGPQA
NLHDACQGDSGGPLVCLNDGRMTLVGIISWGLGCGQKDVPGVYTKVTNYLDWIRDNMRP
