# Supplementary figures and images for: Reteplase Fc-fusions produced in N. benthamiana are able to dissolve blood clots ex vivo
Source: PLoS One. 2021 Nov 30;16(11):e0260796. doi: 10.1371/journal.pone.0260796 (PMC8631678; doi:10.1371/journal.pone.0260796)

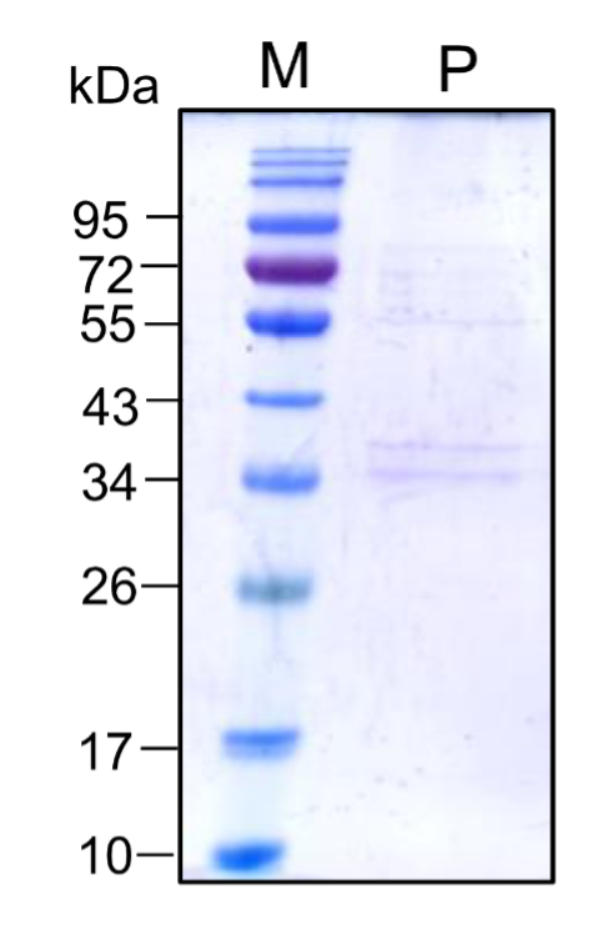

Supplement: S2 Fig — Protein size markers are shown in kilo Dalton (kDa). (TIFF) [file pone.0260796.s002.tiff]

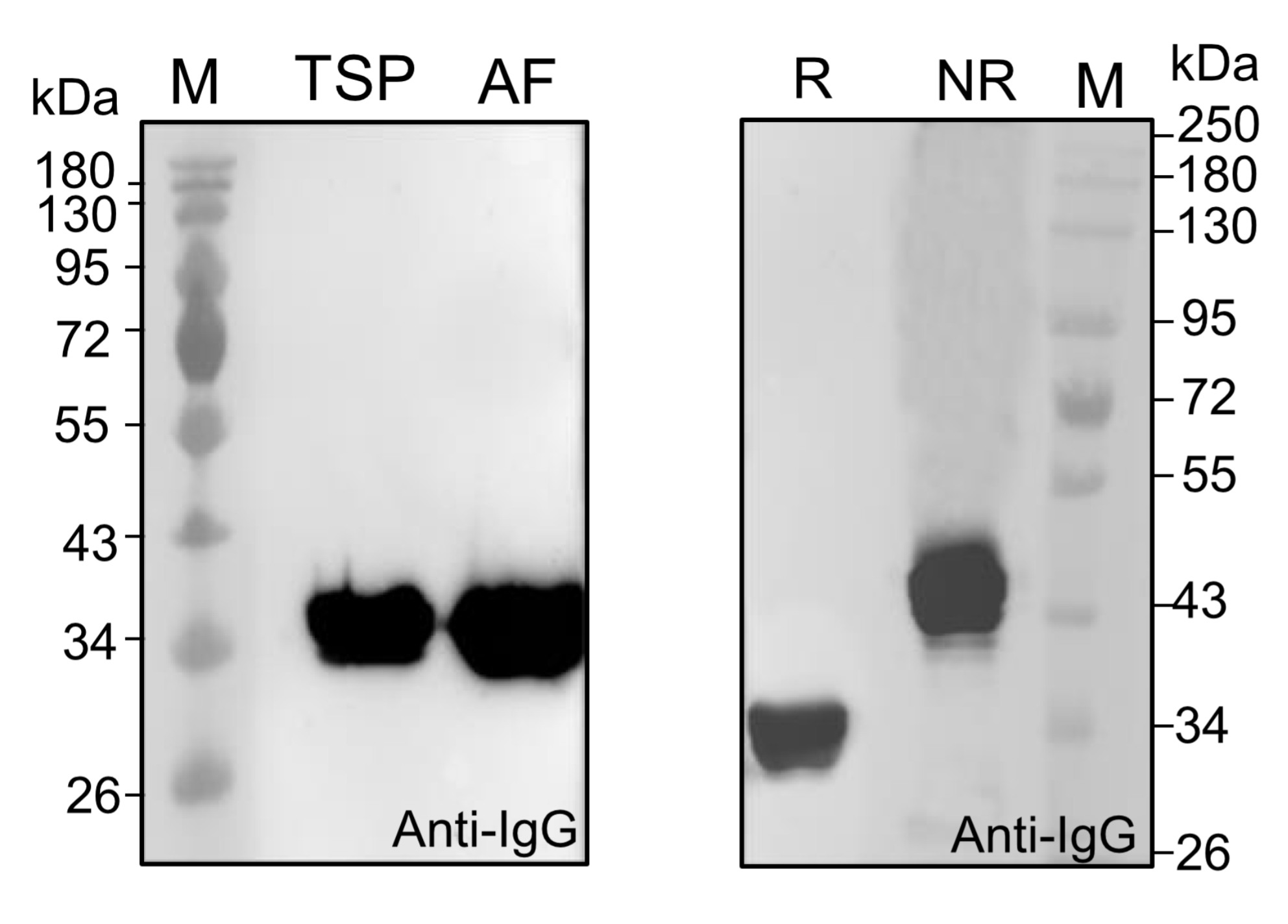

Supplement: S3 Fig — Total soluble proteins extracts (TSP) and apoplastic fluid (AF) from N. benthamiana leaves expressing LFc (fusion partner) were analysed by immunoblotting with anti-IgG antibodies (left panel). TSP were also analysed under reducing and non-reducing conditions with anti-IgG antibodies (right panel). Protein size markers are shown in kilo Dalton (kDa). (TIFF) [file pone.0260796.s003.tiff]

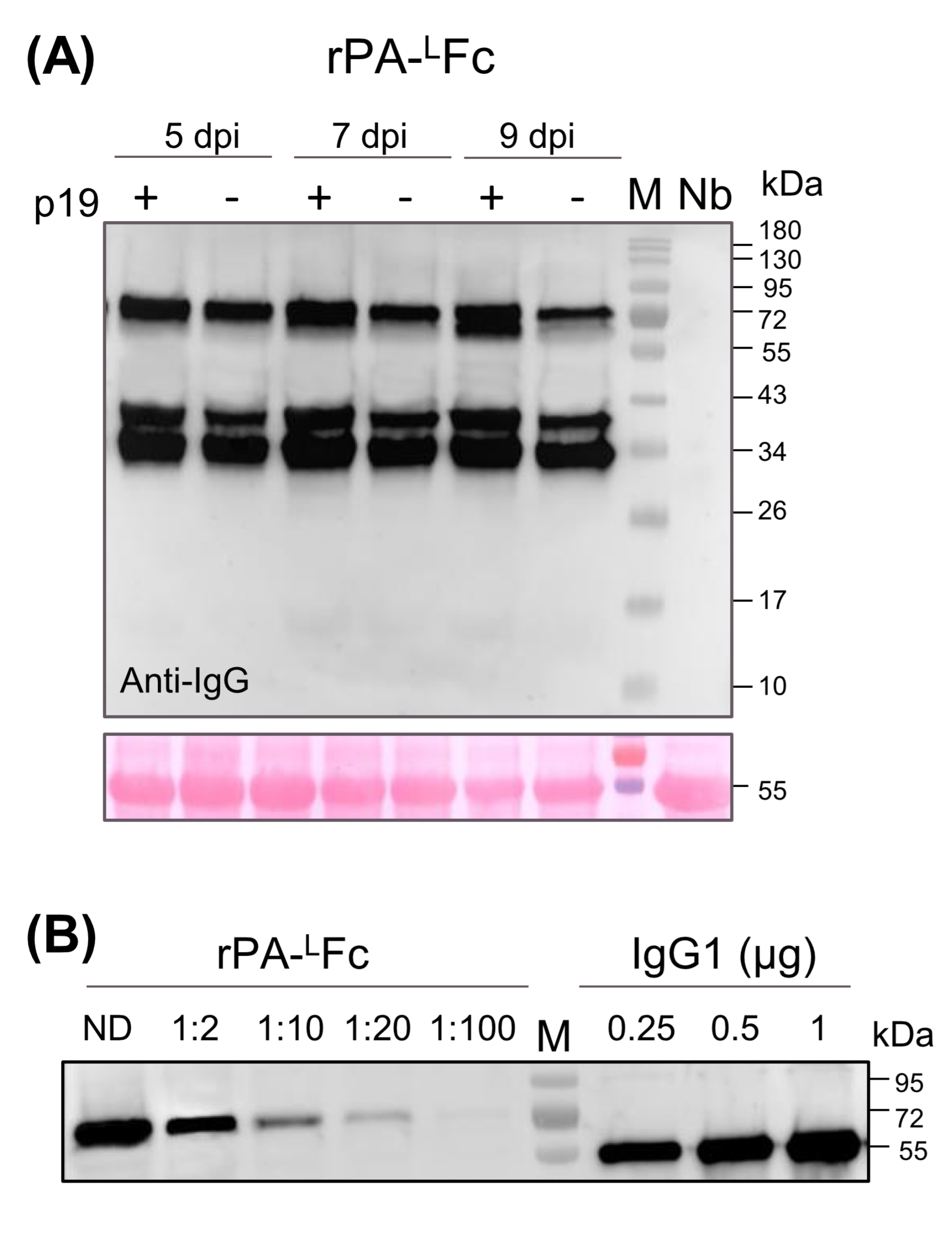

Supplement: S4 Fig — a Total soluble proteins extracts (TSP) from N. benthamiana leaves expressing rPA-LFc with (+) or without (-) co-expression of p19 were analysed by immunoblotting with anti-IgG antibodies at 5-, 7- and 9-days post infiltration (dpi). b Expression of full-length rPA-LFc (72-kDa) in TSP was estimated by a semi‐quantitative western blot analysis with anti-IgG antibodies using IgG1 as standard. Protein size markers are shown in kilo Dalton (kDa). (TIFF) [file pone.0260796.s004.tiff]

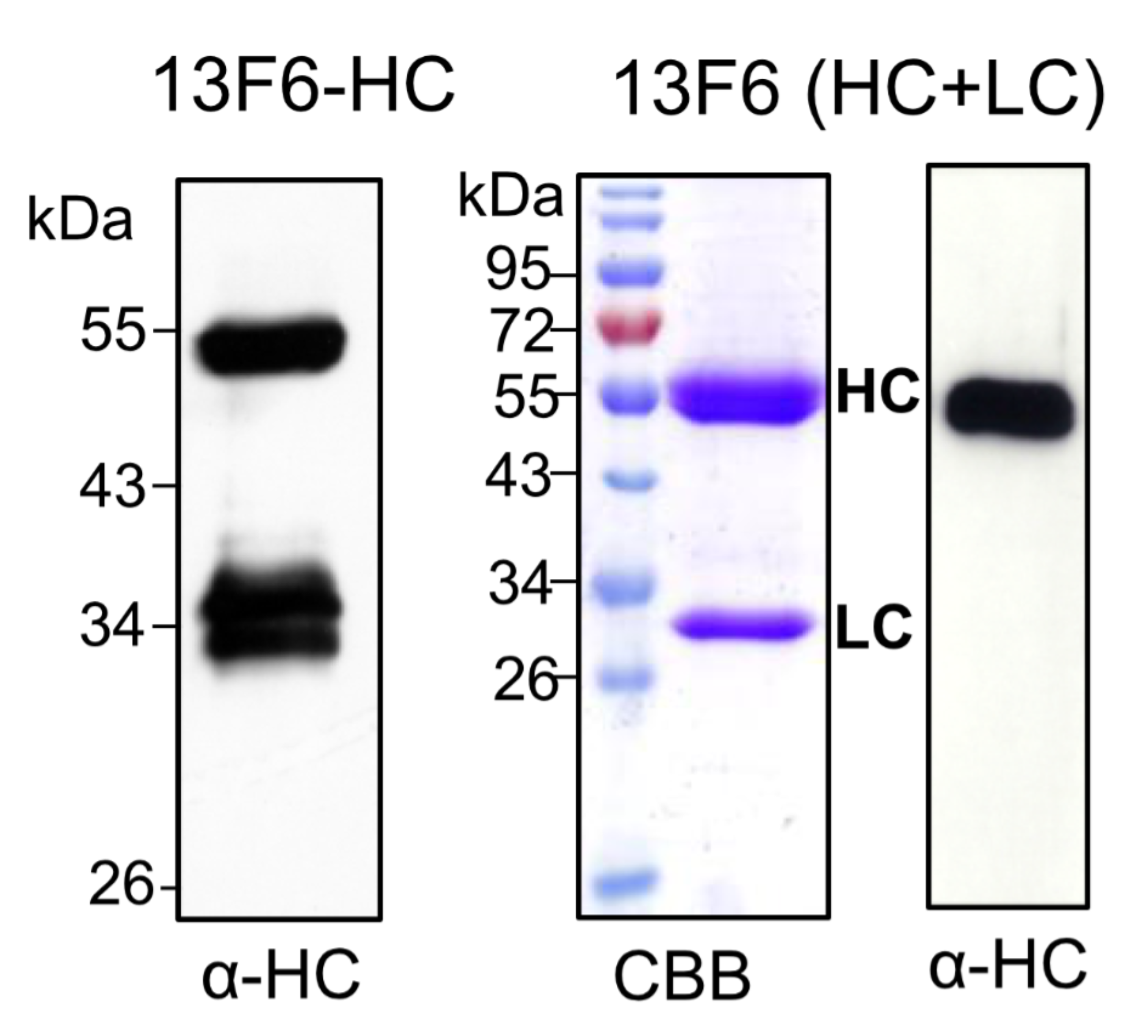

Supplement: S5 Fig — Total soluble proteins extracts (TSP) from N. benthamiana leaves expressing the heavy chain (HC) of the 13F6 monoclonal antibody were analysed by immunoblotting with anti-γ chain antibodies (HC of IgG1). Full 13F6 antibodies (HC and LC) purified out of TSP with protein A were either stained with Commassie brilliant blue (CBB) or subjected to immunoblotting with anti-γ chain antibodies. Protein size markers are shown in kilo Dalton (kDa). (TIFF) [file pone.0260796.s005.tiff]

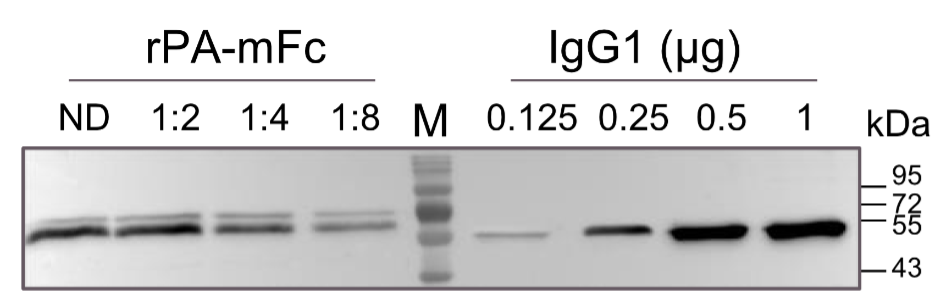

Supplement: S6 Fig — Expression of rPA-mFc (72-kDa) in TSP was estimated by a semi‐quantitative western blot analysis with anti-IgG antibodies using IgG1 as standard. Protein size markers are shown in kilo Dalton (kDa). (TIFF) [file pone.0260796.s006.tiff]
