# Supplementary material for: Reteplase Fc-fusions produced in N. benthamiana are able to dissolve blood clots ex vivo
Source: PLoS One. 2021 Nov 30;16(11):e0260796. doi: 10.1371/journal.pone.0260796 (PMC8631678; doi:10.1371/journal.pone.0260796)
Supplement: S1 Table — XbaI (TCTAGA) BamHI (GGATCC) BsaI (GGTCTC) Eps3I (CGTCTC) restriction sites are in bold. Single-strand DNA overhangs are underlined. (PDF) [file pone.0260796.s008.pdf]

Construct	Primers
rPA-mRFP	rPA F1: TATA**TCTAGA**TCTTACCAGGGCAACAGCGATTGC
	rPA R1: TATA**GGATCC**AGGCCGCATGTTGTCCCTGATCC
rPA-^{H}Fc	HFc F1: GAG**CGTCTC**TAGGTA**GAGACC**GGGATCC**GGTCTC**TGAGCCTAAGTCCTGTGATAAGACTC
	Fc R1**:** GCA**CGTCTC**AAAGCTCACTTTCCAGGAGAAAGAG
	rPA F2: TATA**GGTCTC**AAGGTTCTTACCAGGGCAACAGCGATTGC
	rPA R2: TATA**GGTCTC**AGCTCAGGCCGCATGTTGTCCCTGATCC
rPA-^{L}Fc	LFc F1: GAG**CGTCTC**TAGGTA**GAGACC**GGGATCC**GGTCTC**TGGAGGTGGTGGTTCTGGTGGTGGTGGTTCACTTGGCGGACCATCTG
	Fc R1**:** GCA**CGTCTC**AAAGCTCACTTTCCAGGAGAAAGAG
	rPA F2: TATA**GGTCTC**AAGGTTCTTACCAGGGCAACAGCGATTGC
	rPA R3: TATA**GGTCTC**ACTCCAGGCCGCATGTTGTCCCTGATCC
rPA-mFc	rPA F2: TATA**GGTCTC**AAGGTTCTTACCAGGGCAACAGCGATTGC
	mFc R1: GGTTCTCTAGGTTGACCCTTGGCTTTGGAAATAG
	mFc F1: CCAAAGCCAAGGGTCAACCTAGAGAACCTCAAGTGTAC
	Fc R1**:** GCA**CGTCTC**AAAGCTCACTTTCCAGGAGAAAGAG

Table S1: Primers used in PCR for gene cloning and construction of rPA fusion variants. *Xba*I (TCTAGA) *Bam*HI (GGATCC) *Bsa*I (GGTCTC) *Eps*3I (CGTCTC) restriction sites are in bold. Single-strand DNA *overhangs* are underlined
