# Supplementary material for: Reteplase Fc-fusions produced in N. benthamiana are able to dissolve blood clots ex vivo
Source: PLoS One. 2021 Nov 30;16(11):e0260796. doi: 10.1371/journal.pone.0260796 (PMC8631678; doi:10.1371/journal.pone.0260796)

We present all raw blots and gel image with annotated loading order and experimental samples (in red). Lanes not included in the final figure are marked with an "X" . Dashed lines mark where the blots/gels were cropped to do figures. Images were captured with Fusion Solo S image system (Vilber Lourmat) and Affinity photo was used to enhance contrast/brightness of image.

Figure 4A

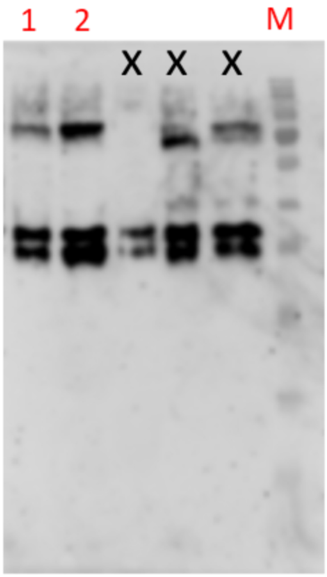

Figure 4B left panel

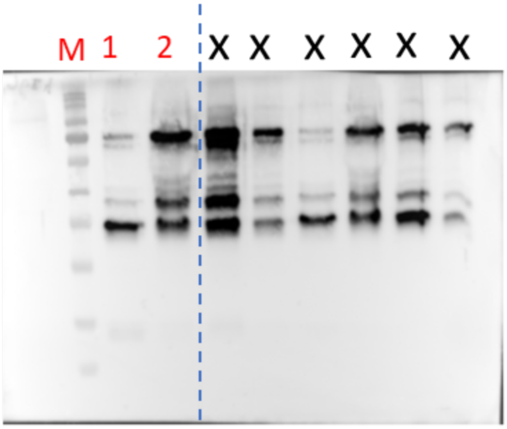

Figure 4B right panel

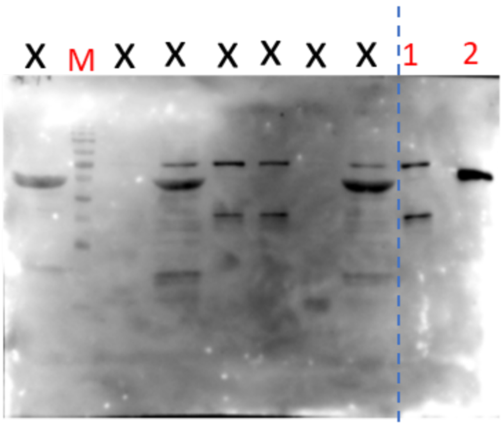

Figure 4C

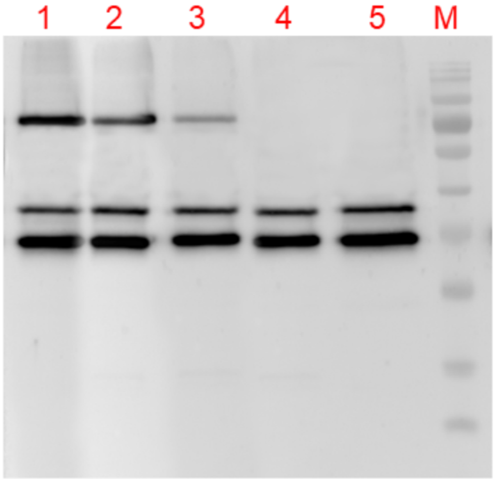

Figure 4D left panel

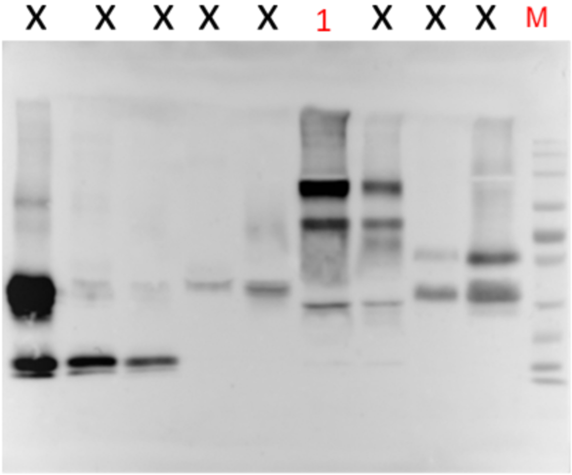

Figure 4D right panel

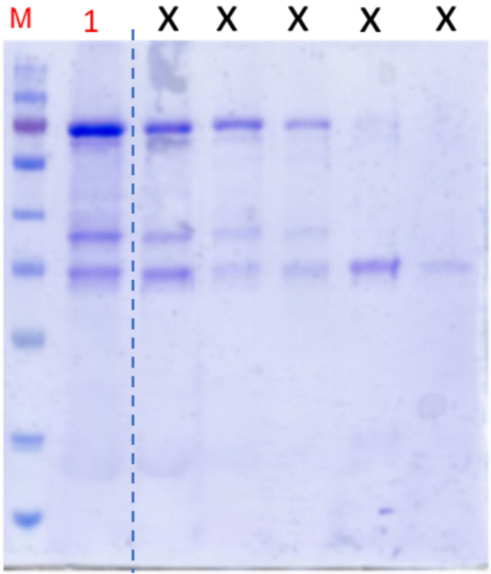

Figure 5A

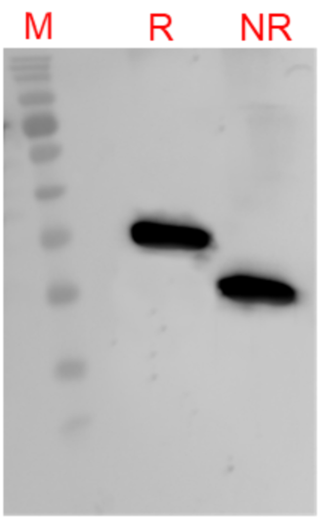

Figure 5B (upper panel)

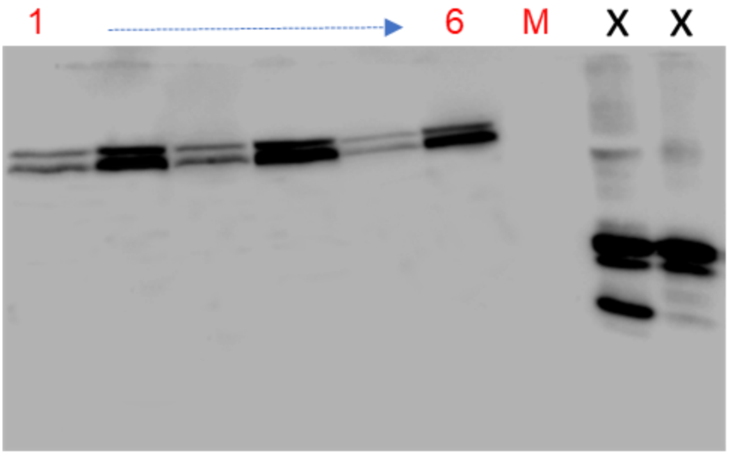

Figure 5C

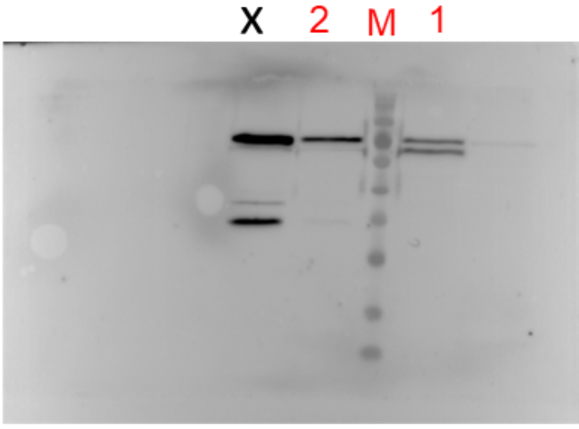

Figure 5B (lower panel)

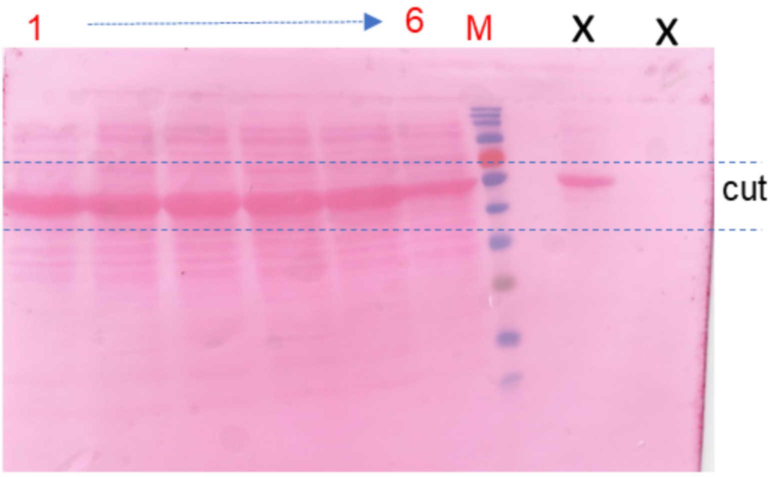

Figure 5D left

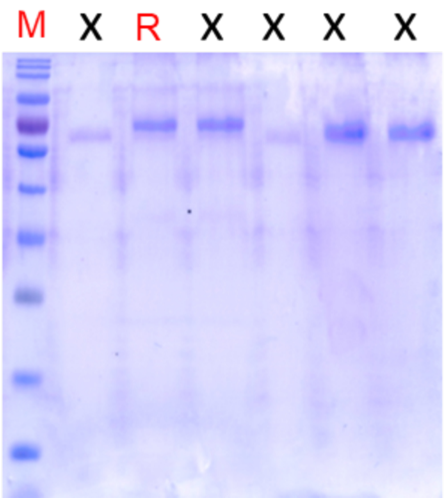

Figure 5D right

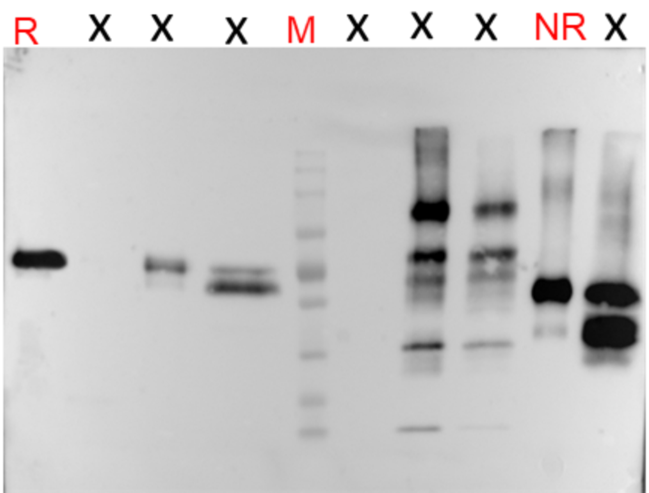

Figure 6A

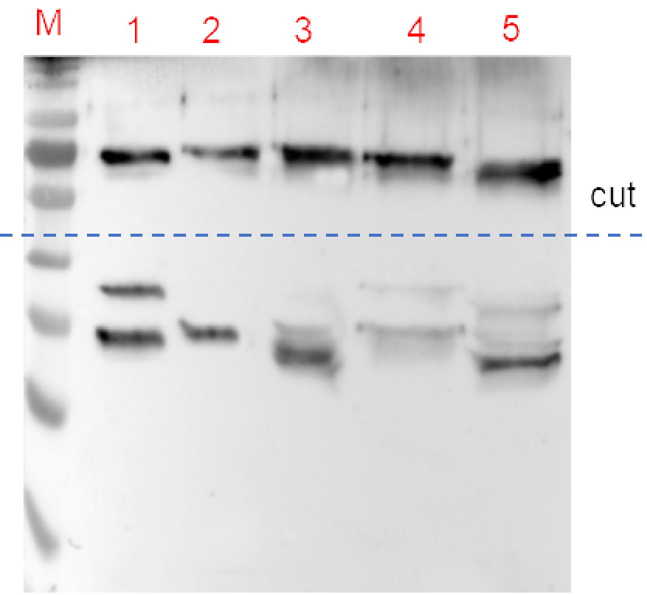

Figure 6B

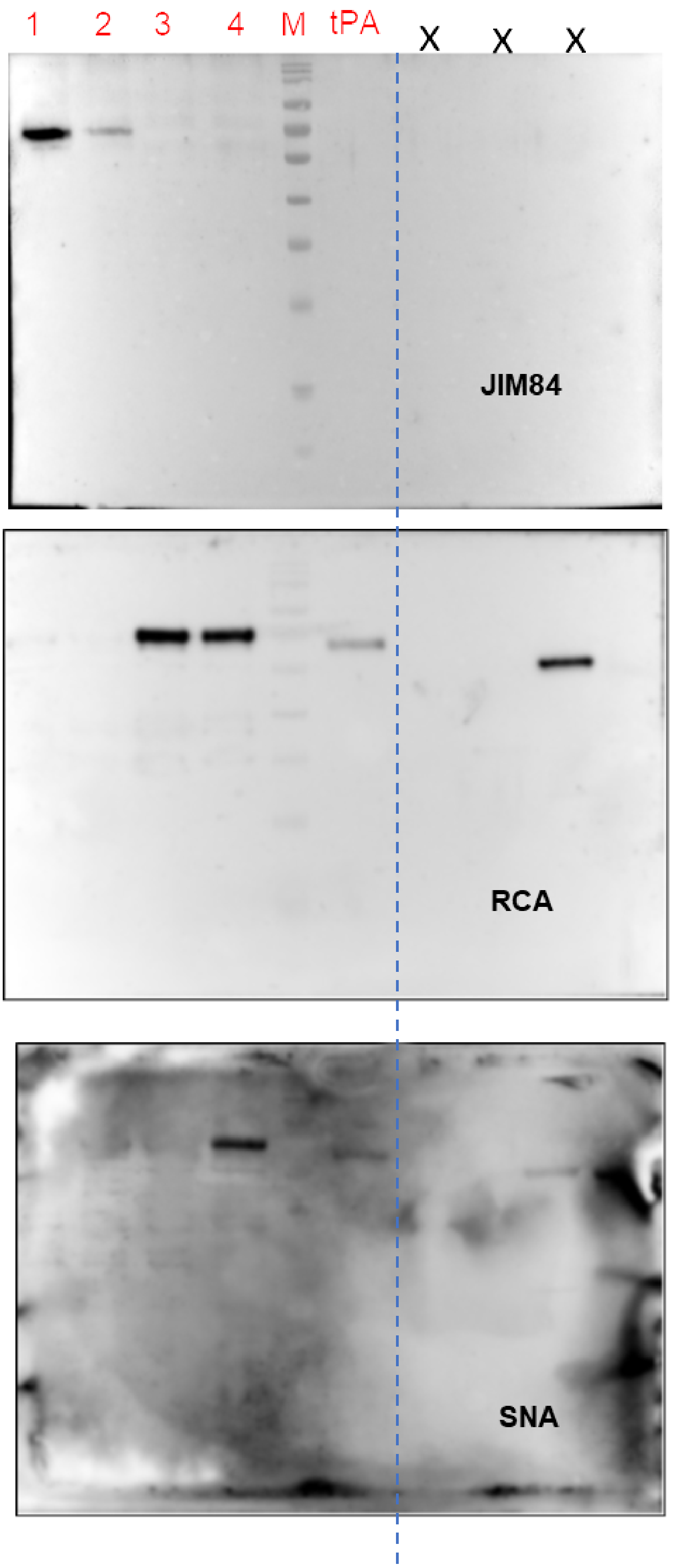

Supplementary Figures

Figure S2

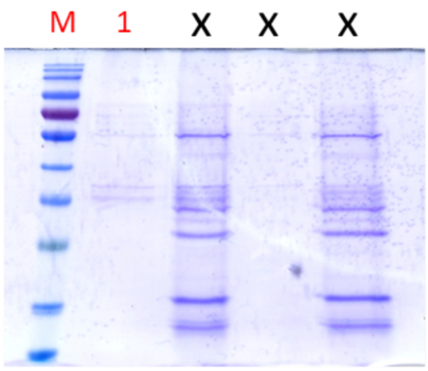

Figure S3 (left panel)

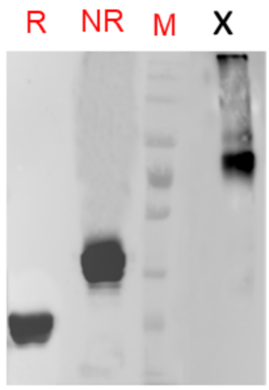

Figure S3 (right panel)

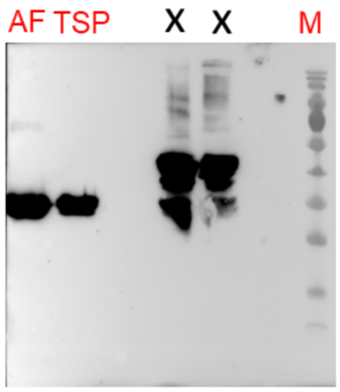

Figure S4A (upper panel)

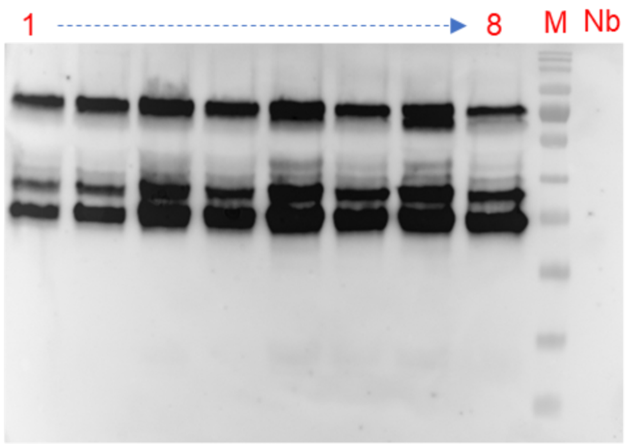

Figure S4A (lower panel)

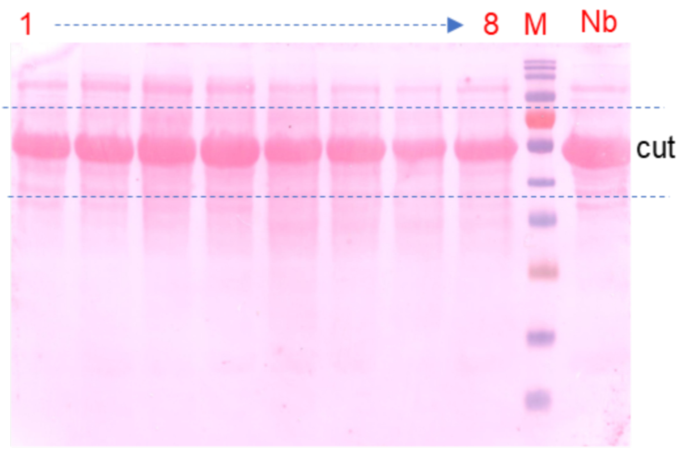

Figure S4B

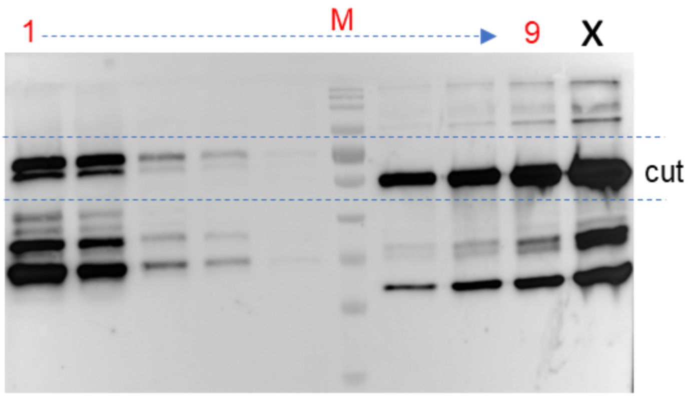

Figure S5

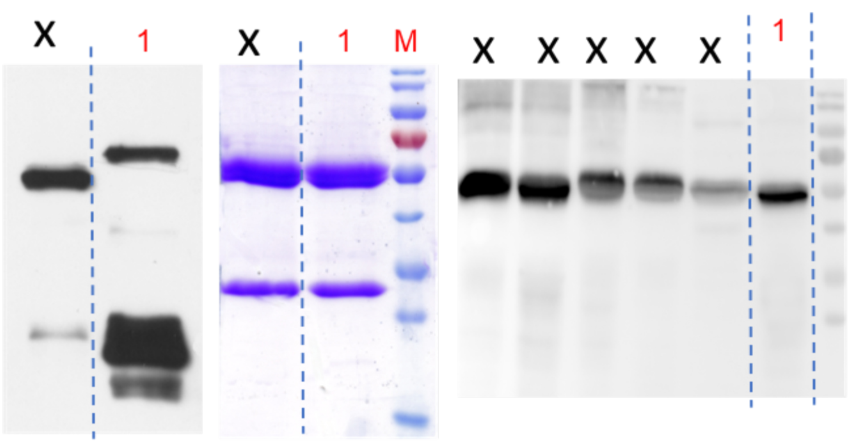

Figure S6

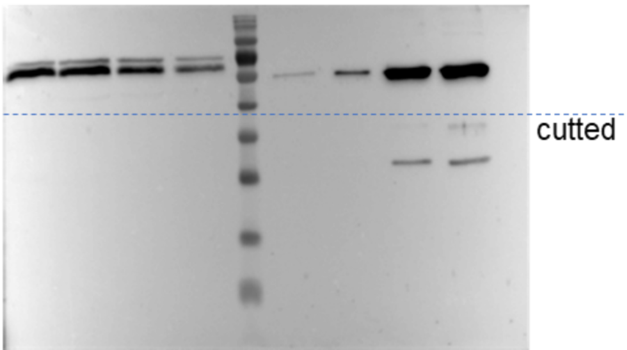

Supplement: S1 Raw images — (PDF) [file pone.0260796.s009.pdf]
